# Increased accrual of diverse patient populations in oncology phase I clinical trials at the University of Colorado Cancer Center

**DOI:** 10.3389/fonc.2025.1546500

**Published:** 2025-07-15

**Authors:** Ahmed Alsafar, Sama L. Kareem, Bradley R. Corr, Christopher H. Lieu, Breelyn Wilky, S. Lindsey Davis, D. Ross Camidge, Antonio Jimeno, Wells A. Messersmith, Andrew Nicklawsky, Daniel Pacheco, Evelinn A. Borrayo, Jessica D. McDermott, Jennifer R. Diamond

**Affiliations:** ^1^ School of Medicine, University of Colorado Anschutz Medical Campus, Aurora, CO, United States; ^2^ Division of Gynecologic Oncology, Department of Obstetrics & Gynecology, University of Colorado Anschutz Medical Campus, Aurora, CO, United States; ^3^ Division of Medical Oncology, Department of Medicine, University of Colorado Anschutz Medical Campus, Aurora, CO, United States; ^4^ University of Colorado Cancer Center Biostatistics Core, University of Colorado Anschutz Medical Campus, Aurora, CO, United States; ^5^ University of Colorado Cancer Center Office of Community Outreach and Engagement, University of Colorado Anschutz Medical Campus, Aurora, CO, United States

**Keywords:** clinical trials, phase I, cancer, social determinants of health, health disparities

## Abstract

**Background:**

Disparities in cancer outcomes persist between racial, ethnic, and socioeconomic groups. One potential cause is lack of appropriate representation in dose-finding clinical trials. We investigated the extent of disparities in phase I clinical trials and recent changes in the setting of institutional efforts to mitigate disparities, legislative interventions, FDA guidance for sponsors and the COVID-19 pandemic.

**Methods:**

We performed a retrospective review of patients enrolled in phase I clinical trials at the University of Colorado Cancer Center in 2018–2019 and 2022-2023. We collected demographics, area deprivation index (ADI), tumor type and other clinical variables. Differences between cohorts were evaluated with t-tests, chi-Square test, or Fisher exact test. Progression-free survival (PFS) and overall survival (OS) were calculated using the Kaplan-Meier method. Hazard ratios (HR), confidence intervals (CI) and p-values were derived using the Cox-proportional hazards method.

**Results:**

A total of 361 patients were included (209 and 152 in the 2018–2019 and 2022–2023 cohorts, respectively). The population consisted of 85.0% White, 3.3% Asian, 1.4% Black, 0.3% Native Hawaiian or Pacific Islander and no American Indian/Alaskan Native (AIAN) patients by race, and 9.1% Hispanic by ethnicity. The most common tumor type was colorectal cancer (18.3%). Compared to 2018-2019, we observed increases in non-English speakers from 1.9% (4/209) to 6.6% (10/152) (p = 0.028) and in translated informed consent forms (ICFs) from 1.4% (3/209) to 5.9% (9/152) (p = 0.033) in 2022-2023. There were no significant changes in race, ethnicity, insurance, or tumor type, although there was a moderate increase in Hispanic patients from 8.1% to 10.5%. There were no differences in clinical outcomes by race, ethnicity, or ADI scores in the overall study population. However, in the most common cancer type, colorectal cancer, higher ADI scores were associated with decreased median PFS and OS.

**Conclusion:**

The interventions resulted in an increase in accrual of non-English speaking patients, however, there was not yet a significant change in overall race and ethnicity. Our study confirms poorer outcomes for patients with higher ADI scores. Further research is warranted to understand disparities in clinical trial accrual, and intervention is needed to improve outcomes for disadvantaged patients.

## 1 Introduction

Approximately 41% of men and 39% of women in the United States are diagnosed with invasive cancer in their lifetime (1). Due to increased awareness, access to early screening and more efficacious treatments, both cancer incidence and cancer death rates have dropped since the early 1990s (1). However, not all populations in the United States have benefited equally from this progress. For example, the relative risk of death is 33% higher in non-Hispanic Black patients and 51% higher in non-Hispanic AIAN patients when compared to non-Hispanic White patients (2). Additionally, 5-year relative survival in Black patients is lower in 19 of the 23 common cancer sites reported by the American Cancer Society compared to White patients (1).

The disparities are multifactorial in nature, and include biologic factors, social determinants of health such as income, education, and health insurance, as well as access to diagnostic testing, genetic counseling, and guideline-directed care which includes clinical trial enrollment for many patients (1, 3–8).

Clinical trials leading to new drug approvals play a critical role in improving cancer outcomes. Equitable enrollment in early phase and registration clinical trials is important for many reasons, including the well-documented differences in the molecular biology of cancer and susceptibility of patients to drug toxicity according to race and ethnicity. Tumors in certain racial groups often have different molecular drivers (9–12). Examples include certain populations of Asian descent having a higher mutation rate in epidermal growth factor receptor (EGFR) in lung cancers, and Black patients having higher mutation rates in p53 in endometrial cancers (11). Differences in molecular alterations may impact presentation of disease, response to treatment, and prognosis. Additionally, differences in drug metabolism and toxicity are also observed in different racial and ethnic groups (3, 13, 14), such as the higher rates of toxicity requiring dose-reductions in Black or Hispanic patients with gastrointestinal tumors receiving Capecitabine (13).

Effect size and risk prediction may not be reliable if data is extrapolated from an unrepresentative clinical trial population onto a more diverse general population. Therefore, recruiting a diverse clinical trial population is essential to ensuring that the approved dose and schedule for new agents, based on the safety profile observed in clinical trials, is appropriate for a diverse cancer patient population. Some studies have also shown survival benefit to early enrollment in a phase I study (15), which further supports consideration of all patients.

There are a number of barriers to recruiting a representative clinical trial population (3, 16–19). Individual-level barriers include negative patient attitudes towards clinical trials, mistrust in the medical system and higher receptivity to alternative medicine in certain groups (17). There are also systemic barriers including the location of treatment accessible to patients, health insurance inadequacy, transportation and additional financial burden (20).

Despite the above barriers, studies have shown that persons from minority groups have similar willingness to participate in research once presented with the opportunity (18, 21). Clinician biases and misconceptions of persons from minority groups may also contribute to the observed accrual disparities due to perceptions and concerns about health literacy and compliance with strict study protocols (19, 22). Similarly, stringent clinical trial criteria may disproportionally exclude populations with a higher prevalence of comorbidities (23).

At the University of Colorado Cancer Center, a set of institutional initiatives were implemented in January of 2022 and included establishment of a bicultural and bilingual Spanish-speaking clinic team, partnerships with the local county hospitals, multi-lingual patient education materials, internal reviews and site assessments. Similarly, initiatives at the legislative level were also implemented both locally (24) and nationally with an FDA guidance for industry sponsors (25).

In the current study, we examined clinical trial participation demographics and treatment outcomes of patients enrolled in early phase clinical trials in two different timeframes to evaluate differences following efforts at the institutional, local and national level to increase diversity.

## 2 Materials and methods

### 2.1 Study design and participants

We performed a retrospective cohort study including patients enrolled in phase I clinical trials at the University of Colorado Cancer Center from 2018–2019 and 2022–2023 to determine the impact of recent efforts on patient demographics and treatment outcomes. If patients were enrolled in multiple phase I studies during the study period, only the first study enrollment was included. Study participants enrolled in the years 2020–2021 were excluded due to the impact of the COVID-19 pandemic on all aspects of clinical research during that time. This was also a time of transition in developing diversity initiatives.

### 2.2 Data collection and storage

Patient demographics and clinical characteristics collected include age, sex, race, ethnicity, language, primary insurance coverage, ADI, pre-treatment body mass index (BMI), Eastern Cooperative Oncology Group (ECOG) performance scores, smoking history, tumor type, clinical trial treatment type, dates and response.

All patient and clinical trial data was collected under an Institutional Review Board (IRB) approved protocol. Patient data was collected from electronic medical records and stored in a secure, password-protected electronic data capture system, RedCap. Data was collected until patient death, loss to follow up, or the data collection cutoff date 8/1/2023, whichever was earlier.

### 2.3 Statistical analysis

Patient characteristics were summarized by cohort into Cohort 1: 2018–2019 and Cohort 2: 2022–2023 using standard descriptive statistics. The differences between cohorts were evaluated with t-tests for continuous variables, the chi-square test for categorical variables, and the Fisher exact test for categorical variables with low cell counts. Median PFS and OS were calculated using the Kaplan-Meier method. Censoring occurred either at the instance of lost to follow-up or using the data cut-off date of 8/1/2023. Hazard rates and their associated p-values were derived using Cox-proportional hazards models. Additional exploratory analysis was performed on select variables and their relationship to PFS and OS. Candidate variables were assessed for multicollinearity, proportional hazards, and the best fitting model was selected utilizing the Akaike information criterion. Significance level, α, was set to 0.05. Data preparation and statistical analyses were conducted using SAS 9.4 (SAS Institute; Cary, NC), and plots were generated using R version 4.2.0 (R Core Team).

## 3 Results

### 3.1 Patient characteristics

A total of 361 patients were included (209 in the 2018–2019 Cohort 1 and 152 in the 2022–2023 Cohort 2). Baseline patient characteristics are shown in **Tables 1** and **2**. The most common tumor type was colorectal cancer (18.3% of the overall study population).

**Table 1:** Patient clinical characteristics.

	Cohort 1: 2018-2019 (n = 209)	Cohort 2: 2022-2023 (n = 152)	P-value	Both cohorts (n = 361)
Age				
Mean (SD) Median (IQR)	59.83 (11.89) 60.12 (52.61, 68.56)	60.52 (12) 61.66 (51.85, 69.78)	0.588 ^a^	60.12 (11.92) 60.94 (52.44, 69.29)
Sex			0.736 ^b^	
Female	111 (53.1%)	78 (51.3%)		189 (52.4%)
Male	98 (46.9%)	74 (48.7%)		172 (47.7%)
ECOG Performance Status			0.941 ^c^	
0	90 (43.1%)	68 (44.7%)		158 (43.8%)
1	116 (55.5%)	82 (54.0%)		198 (54.9%)
2	3 (1.4%)	2 (1.3%)		5 (1.4%)
Smoking Status			0.787 ^b^	
Current Smoker	7 (3.4%)	6 (4.0%)		13 (3.6%)
Non-smoker	115 (55.0%)	88 (57.9%)		203 (56.2%)
Past Smoker	87 (41.6%)	58 (38.2%)		145 (40.2%)
BMI			0.821 ^b^	
<18.5	16 (7.7%)	12 (7.9%)		28 (7.8%)
18.5 - 24.9	90 (43.1%)	59 (38.8%)		149 (41.3%)
25 - 29.9	66 (31.6%)	49 (32.2%)		115 (31.9%)
≥30	37 (17.7%)	32 (21.1%)		69 (19.1%)
Tumor Type			0.355 ^b^	
Gastrointestinal	78 (37.3%)	61 (40.1%)	0.228 ^b,d^	139 (38.5%)
Colorectal	32 (15.3%)	34 (22.4%)		66 (18.3%)
Pancreatic	29 (13.9%)	14 (9.2%)		43 (11.9%)
Gastroesophageal	8 (3.8%)	4 (2.6%)		12 (3.3%)
Bile Duct/Gallbladder	4 (1.9%)	2 (1.3%)		6 (1.7%)
Other Gastrointestinal	5 (2.4%)	7 (4.6%)		12 (3.3%)
Gynecologic	25 (12.0%)	21 (13.8%)		46 (12.7%)
Sarcoma	26 (12.4%)	18 (11.8%)		44 (12.2%)
Breast	26 (12.4%)	13 (8.6%)		39 (10.8%)
Lung	26 (12.4%)	12 (7.9%)		38 (10.5%)
Head and Neck	13 (6.2%)	19 (12.5%)		32 (8.9%)
Skin	6 (2.9%)	3 (2.0%)		9 (2.5%)
Genitourinary	6 (2.9%)	2 (1.3%)		8 (2.2%)
Lymphoma	1 (0.5%)	0 (0%)		1 (0.3%)
Other	2 (1.0%)	3 (2.0%)		5 (1.4%)
Clinical Trial Treatment Type			0.01 ^b^	
Immunotherapy	103 (49.3%)	84 (55.3%)		187 (51.8%)
Targeted Therapy	64 (30.6%)	40 (26.3%)		104 (28.8%)
Antibody-drug Conjugate	8 (3.8%)	15 (9.9%)		23 (6.4%)
Cytotoxic Therapy	8 (3.8%)	0 (0.0%)		8 (2.2%)
Combinations of Different Treatment Types	26 (12.44%)	13 (8.6%)		39 (10.8%)

SD, standard deviation; IQR, interquartile range; ECOG, Eastern Cooperative Oncology Group; BMI, body mass index.

a T-Test.

b Chi-square Test.

c Fisher Exact Test.

d Gastrointestinal tumor type-specific p-value.

**Table 2:** Patient social determinants of health.

	Cohort 1: 2018-2019 (n = 209)	Cohort 2: 2022-2023 (n = 152)	P-value	Both cohorts (n = 361)
Race				
White	176 (84.2%)	131 (86.2%)	0.279 ^a^	307 (85.0%)
Asian	7 (3.4%)	5 (3.3%)		12 (3.3%)
Black or African American	5 (2.4%)	0 (0%)		5 (1.4%)
More Than One Race	1 (0.5%)	3 (2.0%)		4 (1.1%)
Native Hawaiian or Other Pacific Islander	1 (0.5%)	0 (0%)		1 (0.3%)
Unknown/Not Reported	19 (9.1%)	13 (8.6%)		32 (8.9%)
American Indian/Alaskan Native	0 (0%)	0 (0%)		0 (0%)
Ethnicity				
Not Hispanic or Latino	192 (91.9%)	135 (88.8%)	0.352 ^a^	327 (90.6%)
Hispanic or Latino	17 (8.1%)	16 (10.5%)		33 (9.1%)
Unknown/Not Reported	0 (0%)	1 (0.7%)		1 (0.3%)
Preferred Language (Collapsed)				
English	205 (98.1%)	142 (93.4%)	0.028 ^a^	347 (96.1%)
Other	4 (1.9%)	10 (6.6%)		14 (3.9%)
Preferred Language				
English	205 (98.1%)	142 (93.4%)	0.070 ^a^	347 (96.1%)
Spanish	3 (1.4%)	5 (3.3%)		8 (2.2%)
Bosnian	0 (0%)	1 (0.7%)		1 (0.3%)
Mandarin Chinese	0 (0%)	1 (0.7%)		1 (0.3%)
Mongolian	0 (0%)	1 (0.7%)		1 (0.3%)
Punjabi	1 (0.5%)	0 (0%)		1 (0.3%)
Ukrainian	0 (0%)	1 (0.7%)		1 (0.3%)
Vietnamese	0 (0%)	1 (0.7%)		1 (0.3%)
Translated Consent Use				
No (English ICF was used)	206 (98.6%)	143 (94.1%)	0.033 ^a^	349 (96.7%)
Yes	3 (1.4%)	9 (5.9%)		12 (3.3%)
Health Insurance Status				
Medicare	89 (42.6%)	76 (50.0%)	0.368 ^a^	165 (45.7%)
Private Insurance	101 (48.3%)	60 (39.5%)		161 (44.6%)
Medicaid	18 (8.6%)	15 (9.9%)		33 (9.1%)
Uninsured	1 (0.5%)	1 (0.7%)		2 (0.6%)
ADI ^b^				
1-5	118 (63.4%)	88 (62.4%)	0.849 ^c^	206 (63.0%)
6-10	68 (36.6%)	53 (37.6%)		121 (37.0%)

ICF, informed consent form; ADI, area deprivation index.

a Fisher Exact Test.

b The number of patients with area deprivation index values does not add up to the total number of patients due to 34 patients (23 in Cohort 1 and 11 in Cohort 2) not having ADI scores due to being located outside the state or due to the data not being available for their home address.

c Chi-square Test.

In both cohorts, the majority of patients were White (85.0%) and 9.1% were Hispanic. By comparison, The rates of new cancer cases in Colorado by race and ethnicity are reported in **Table 3** (26). The majority of patients were English-speaking (96.1%) and lived in neighborhoods with ADI scores of 1-5 (63.0%).

**Table 3:** State of Colorado new cancer cases by race and ethnicity (2015–2020).

Race	Number of new cases (% of total)
White	137,102 (92.0%)
Black	5,155 (3.5%)
Asian/Pacific Islander	2986 (2.0%)
American Indian/ Native Alaskan	1,052 (0.7%)
Other/Unknown	2,697 (1.8%)
Ethnicity	Number of new cases (% of Total)
Hispanic	17,482 (11.7%)
Not Hispanic	13,1459 (88.2%)
Unknown	51 (0.0%)

Patients were enrolled in 80 clinical trials (**Supplementary Materials Figure 1** and **Supplementary Data 1**) receiving immunotherapy (51.8%), targeted therapy (28.8%), antibody-drug conjugates (6.4%), cytotoxics (2.2%) or combination therapy (10.8%)(**Table 1**).

### 3.2 Demographic and clinical characteristics of patients in cohorts 1 and 2

Non-English-speaking patients increased from 1.9% in Cohort 1 to 6.6% (p = 0.028) in Cohort 2. The number of translated ICFs also increased from 1.4% to 4.9% (p = 0.033) and included multiple languages (**Table 2**). There were no significant differences in other demographic or clinical characteristics between the cohorts.

### 3.3 Clinical outcomes for patients by race, ethnicity and other social determinants of health

**Figure 1:**
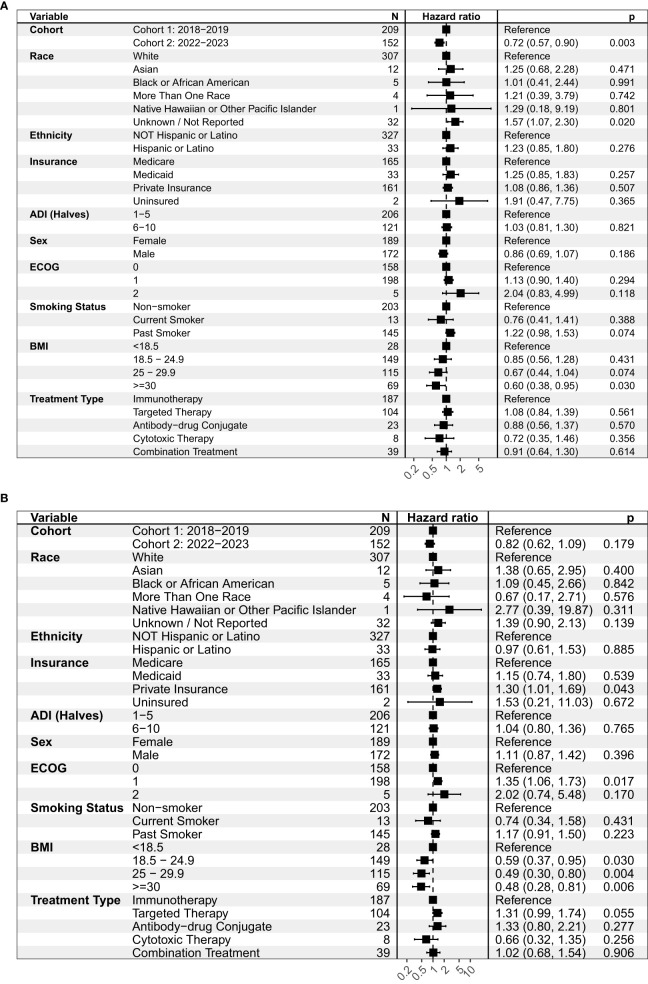
Univariate analysis on **(A)** progression-free survival (PFS) and **(B)** overall survival (OS) for the overall study population (Cohorts 1 and 2). This figure shows the hazard ratio, or the probability of an event such as a progression of disease **(A)** or expiration **(B)** relative to a reference point. ADI, Area deprivation index; ECOG, Eastern Cooperative Oncology Group; BMI, Body mass index.

In the overall study population (Cohorts 1 and 2), we did not observe differences in PFS or OS by patient race or ethnicity (**Figure 1**). Patients with private insurance had a shorter median OS of 7.9 months vs. 9.5 months in patients with Medicare (HR = 1.30, 95% C.I. 1.01-1.69, p = 0.043). Patients with private insurance were younger and more likely to be non-smokers compared to patients with Medicare (**Table 4**). ECOG 1 was associated with shorter OS of 7.8 months vs. 11 months in ECOG 0 (HR = 1.35, 95% CI 1.06-1.73, p = 0.017).

**Table 4:** Selected comparison of patients with Medicare and private insurance.

	Medicare (N = 165)	Private insurance (N = 161)	P-value
Age			<0.001^a^
Mean (SD)	68.44 (8.29)	53.29 (10.21)	
Median (IQR)	69.29 (65.9, 73.63)	54.89 (47.1, 59.91)	
Sex			0.9008^b^
Male	78 (47.27%)	75 (46.58%)	
Female	87 (52.73%)	86 (53.42%)	
Area Deprivation Index			0.9367^b^
1-5	99 (68.28%)	101 (68.71%)	
6-10	46 (31.72%)	46 (31.29%)	
ECOG Performance Status			0.2475^b^
0	67 (40.61%)	79 (49.07%)	
1	95 (57.58%)	81 (50.31%)	
2	3 (1.82%)	1 (0.62%)	
Smoking Status			0.008^c^
Non-smoker	82 (49.7%)	102 (63.35%)	
Past Smoker	80 (48.48%)	52 (32.3%)	
Current Smoker	3 (1.82%)	7 (4.35%)	
BMI			0.287^c^
<18.5	15 (9.09%)	9 (5.59%)	
18.5 - 24.9	71 (43.03%)	64 (39.75%)	
25 - 29.9	53 (32.12%)	51 (31.68%)	
≥30	26 (15.76%)	37 (22.98%)	

SD, standard deviation; IQR, interquartile range; ECOG, Eastern Cooperative Oncology Group; BMI, body mass index.

a T-Test.

b Chi-square Test.

c Fisher Exact Test.

When comparing clinical outcomes of patients between cohorts, the median PFS was 1.9 months in Cohort 1 and 2.8 months in Cohort 2 (HR = 0.72, 95% CI 0.57-0.90, p = 0.003) (**Supplementary Figure 2A**, **Supplementary Table 1**). Median OS was 8.2 months in Cohort 1 and 11.1 months in Cohort 2, although this increase was not statistically significant (HR = 0.82, 95% CI 0.62-1.09, p = 0.179) (**Supplementary Figure 2B**, **Supplementary Table 2**).

**Figure 2:**
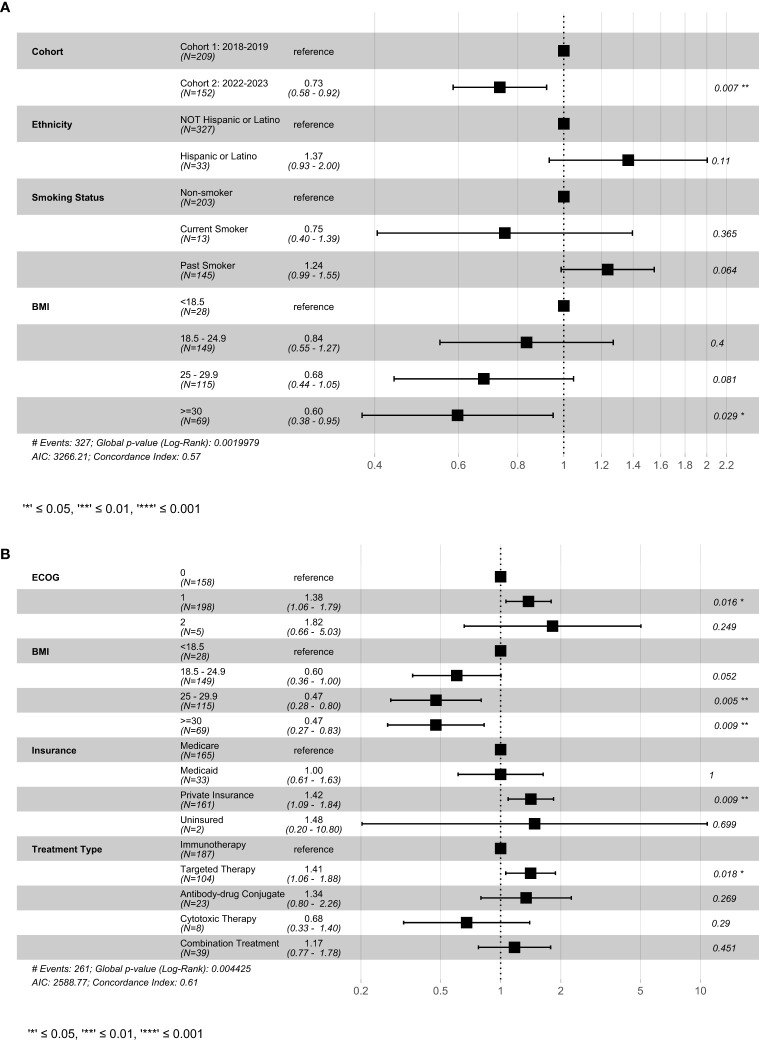
Result of cox-proportional hazard model examining select variables on **(A)** progression-free survival and **(B)** overall survival. This figure shows the best fitting models generated using the stepwise-selection algorithm relying on AIC. Variables were selected based on univariate results in previous figures. BMI, Body mass index; ECOG, Eastern Cooperative Oncology Group; AIC, Akaike information criterion.

In multivariable analysis, enrollment during Cohort 2 and BMI ≥ 25 was associated with improved OS (**Figure 2**). In contrast, ECOG 1, private insurance and treatment with a targeted therapy (versus immunotherapy) were associated with worse OS.

### 3.4 Impact of clinical variables on clinical outcomes in CRC patients

**Figure 3:**
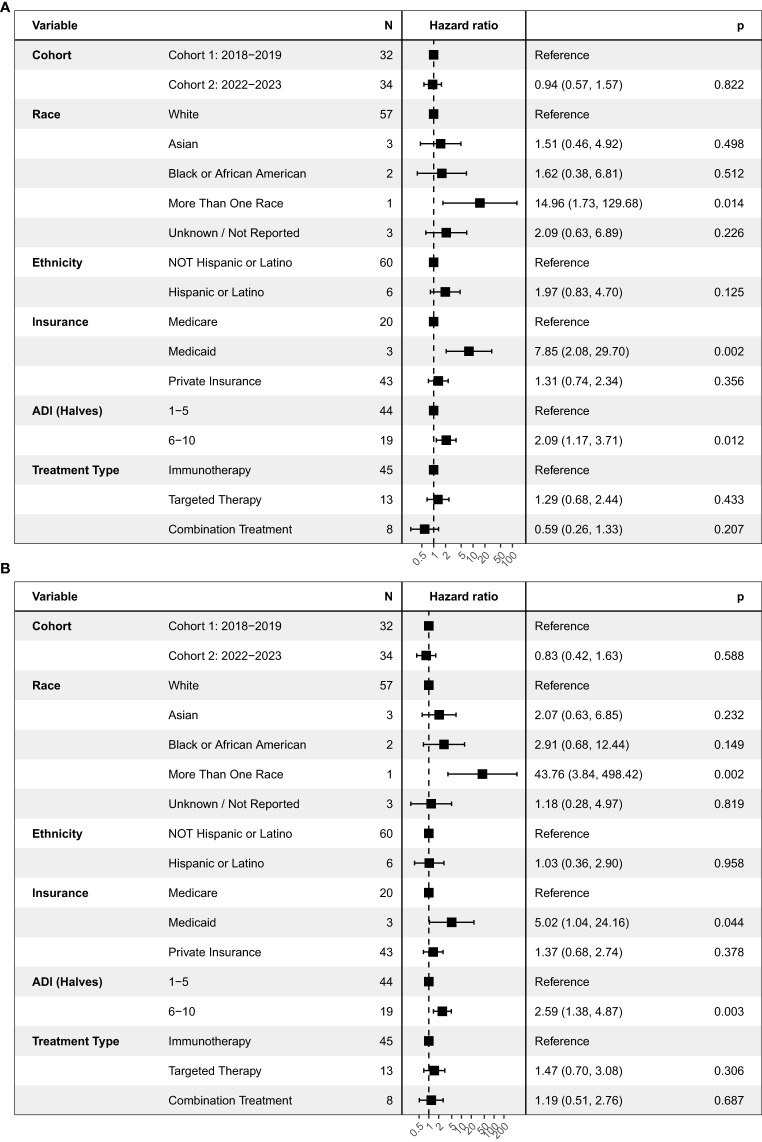
Univariate analysis on **(A)** progression-free survival and **(B)** overall survival in patients with colorectal cancers. This figure shows the hazard ratio, or the probability of an event such as a progression of disease **(A)** or expiration **(B)** relative to a reference point. ADI, Area deprivation index.

We performed an additional exploratory survival analysis in patients with CRC, which was the most common cancer type in our study. Analyzing a single tumor type allowed us to evaluate differences in survival without the known clinical differences between cancer types. In patients with CRC, there was no difference in PFS or OS between Cohorts 1 and 2 (**Figure 3**, **Supplementary Figure 3**). However, ADI scores of 6–10 were associated with worse median PFS of 1.7 months vs 2.8 months in ADI of 1-5 (HR = 2.09, 95% CI 1.17-3.71, p = 0.012) (**Supplementary Table 3**) and OS at 4.2 months vs. 15 months (HR = 2.59, 95% CI 1.38-4.87, p = 0.003) (**Supplementary Table 4**).

**Figure 4:**
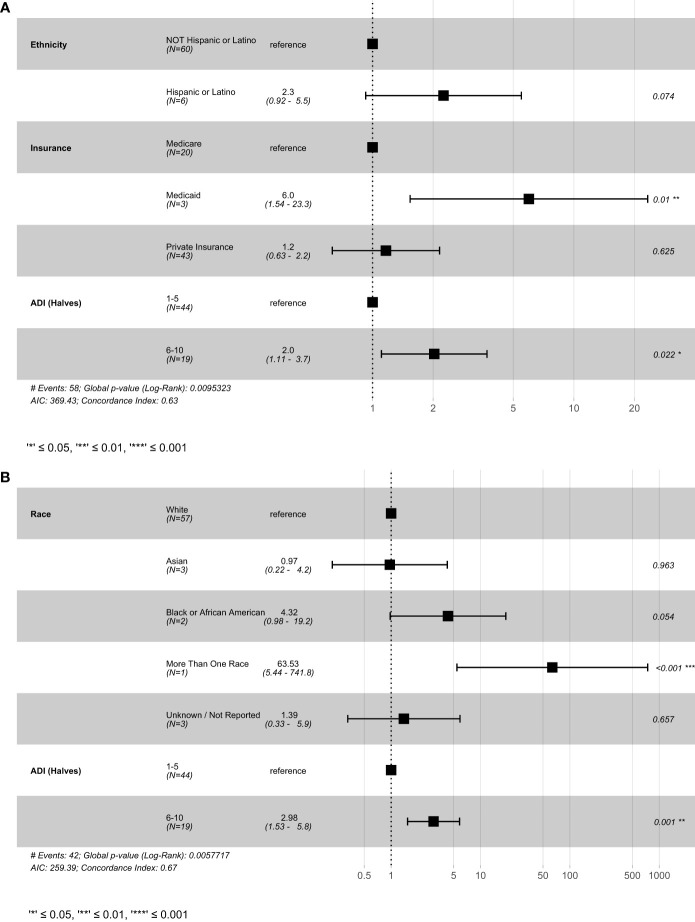
Result of cox-proportional hazard model for colorectal cancer patients **(A)** progression-free survival and **(B)** overall survival. This figure shows the best fitting models generated using the stepwise-selection algorithm relying on AIC. Variables were selected based on univariate results in previous figures. ADI, Area deprivation index; AIC, Akaike information criterion.

Cox multivariable analysis in the CRC subset of patients (**Figure 4**) revealed decreased PFS in patients with Medicaid (p = 0.01), and decreased PFS and OS in patients with ADI scores of 6-10 (PFS; p = 0.022) (OS; p = 0.001) (**Figure 4**).

## Discussion

In this study, we evaluated the demographics and treatment outcomes of patients enrolled in therapeutic anti-cancer phase I clinical trials to assess for changes in the enrollment of underrepresented persons from minority groups. Our study shows several improvements in moving towards a more representative clinical trial population including increases in the number of non-English speakers, the number of translated ICFs and an increase in proportion of Hispanic or Latino patients. Overall, the current findings indicate the success of a range of efforts targeting these populations such as the bicultural clinic, which includes Spanish-speaking providers, research nurses, medical assistants, patient navigators, and scheduling staff, and bilingual patient education materials. Although no significant improvements in other markers of diversity such as race, ethnicity and ADI were seen, the inclusion of several new languages in addition to Spanish supports interventions targeting a broader population, such as the community partnerships through the Office of Community Outreach and Engagement to reach patients at county hospitals as well as quarterly internal reviews and annual presentations to address shortcomings.

Despite widespread efforts dating back to the National Institute of Health Revitalization Act of 1993 (27), representation of persons from minority groups in research has remained inadequate. Recent interventions in the literature have included the use of culturally-competent patient navigators and cultural-competency training, with positive results in different underrepresented populations (28, 29). Another study established a 5-year center-wide program consisting of outreach, marketing, partnerships with local organizations, ride-sharing, nurse navigators, and improved ICFs, which resulted in an increase of Black patients on clinical trials (30), highlighting the efficacy of a multi-faceted and targeted approach. Other interventions that have shown improvements in persons from minority groups outcomes outside of clinical trials have included automated electronic medical record alerts, and frequent institutional reviews of treatment metrics (6).

At our center, we set out to implement a multifaceted set of interventions to address the different barriers known to limit the accrual of historically underrepresented patients to phase I clinical trials and build upon interventions previously shown to be effective at other centers. One of the primary barriers targeted at our center and by FDA guidance for sponsors was the lingual and cultural barrier faced by non-English-speaking patients. Recruiting non-English-speaking patients has always been an institutional logistical challenge, due to added complexity, time and cost required to present clinical trials and translate study documents. Prior studies have shown that this is particularly evident in non-industry-sponsored studies, where the cost of document translation often falls on the investigator team (31). Nonetheless, the improvement in representation of this group suggests that this is a modifiable barrier.

In addition, our study also showed an increase in the percentage of Hispanic or Latino patients from 8.1% to 10.5%. As previously noted, this group of patients made up 9.8% of recent cancer diagnoses in the state, which indicates an appropriate current rate of clinical trial participation. However, the Hispanic or Latino demographic makes up 22.5% of the state’s population (32). The discrepancy between new cancer cases in the state and general population rates is likely due to the younger age of the Hispanic or Latino population in the state (32) as well as the lower cancer screening rates in this population (33). Lastly, changes seen in our study demographics may also reflect recent migration patterns and changes in the state demographics.

Our findings also demonstrate persistent disparities in research. For example, the ADI distribution showed that a majority of patients (63.0%) were placed in the 5 lower deprivation deciles which correspond to higher income, education, employment, housing quality among other measures of affluency (34). This may be due to more affluent patients having better access to cancer screening, diagnosis, treatment at a tertiary center and subsequently access, eligibility and willingness to phase I clinical trials. Additionally, longer distances between a patient’s residence to the treatment center, which may correlate with higher deprivation scores, likely confound the lower participation rates due to the nature of phase I clinical trials, which often require patients to travel to the treatment center multiple times a week. Interestingly, private insurance was associated with worse overall survival compared to Medicare, which may reflect tumor latency in the older Medicare population.

Discrepancies in race-reporting practices between different databases and research studies have long hindered health equity research, and consistency in methodology, improvements to current racial and ethnic categories and potentially implementing the ancestry system to better group individuals of a common lineage (35) would facilitate more accurate representation and comparisons between different sources (36).

Additionally, our clinical trial population consisted of only 0.6% uninsured patients, who make up 6.5% of the state’s population (37). It is likely that the lack of insurance limits their access to cancer screening, diagnosis, and treatment. Recent state-level legislature aims to address this barrier by reducing costs of care (24).

One of the key challenges in assessing survival was the heterogeneity of our study population, which included a wide range of tumor types, treatments, and demographics. Nonetheless, survival analysis was performed to explore overall trends. We noted an improvement in PFS in the 2022–2023 cohort, which likely reflects changes in patient composition as well as clinical trial treatments. To further assess contributions of different social factors on patient outcomes, we analyzed a smaller subset of patients with colorectal cancers. We noted worse outcomes, as shown by both PFS and OS in patients with Medicaid and patients living in areas with higher ADI scores. This association of worse outcomes with higher ADI scores has been well documented across a number of tumor types (38–41) and in phase II and III clinical trials sponsored by the SWOG Cancer Research Network (42). To our knowledge, this is the first study reporting this association across a number of phase I clinical trials However, most phase I clinical trials are not designed to evaluate efficacy outcomes, so the relevance of our observation is limited.

Beyond clinical trials, the advancement of precision medicine and multi-omics datasets has led to the identification of genetic variants that influence complex diseases and drug responses, with important differences observed between populations (43). While diversity in large datasets enhances generalizability and equity, increased heterogeneity can introduce greater variability, necessitating larger sample sizes and added statistical power to reliably detect inter-population differences. The development of advanced analytical methodologies, such as the incorporation of local ancestry, polygenic risk scores, expression quantitative trait locus mapping, and transcriptome-wide association studies, may aid in interpreting these variants in the absence of matched population reference panels, particularly in admixed populations (43).

Our study period overlapped with the COVID-19 pandemic, which had a significant impact on the healthcare system as a whole, including accrual to and conduct of oncology clinical trials (8, 44–48). Other limitations of the current study include the small sample size and single-intuition design, which may limit generalizability of the data to other regions and institutions.

In conclusion, improving the diversity of patients in phase I and registration clinical trials continues to be of utter importance to determine appropriate efficacy, dosage, the detection of accurate toxicity of new treatments. Further efforts to address the poor accrual and clinical outcomes of disadvantaged populations are warranted. To address the persistent poor accrual of some disadvantaged populations seen in our study, future efforts may include more inclusive clinical trial designs (49), and a push towards decentralizing clinical trials to combat the distance and cost barriers (50). Additionally, state-level legislature may also encourage the inclusion of underinsured patients, similar to a bill recently passed in the state of Colorado (24). The impact of such legislation remains to be seen. Lastly, improvements to race, ethnicity reporting, including standardization, grouping patients by ancestry and advancements in multi-omics may help guide future interventions (35, 43).

## Data Availability

The raw data supporting the conclusions of this article will be made available by the authors, without undue reservation.
