# Supplementary material for: Increased accrual of diverse patient populations in oncology phase I clinical trials at the University of Colorado Cancer Center
Source: Front Oncol. 2025 Jul 15;15:1546500. doi: 10.3389/fonc.2025.1546500 (PMC12303809; doi:10.3389/fonc.2025.1546500)
Supplement: Supplementary file 1 [file DataSheet1.docx]

**Supplementary Materials:**

Supplementary Figure 1: Phase 1 Clinical Trials Patient Diagram

Supplementary Figure 2: (A) Progression-free Survival by Cohort (B) Overall Survival by Cohort (Kaplan-Meier curve)

Supplementary Figure 3: (A) Progression-free Survival by Cohort in CRC patients and (B) Overall Survival by Cohort in CRC Patients (Kaplan-Meier curve)

Supplementary Table 1: Median Progression-free Survival for Select Variables

Supplementary Table 2: Median Overall Survival for Select Variables

Supplementary Table 3: Median Progression-free Survival for Select Variables in Colorectal Cancer Patients

Supplementary Table 4: Median Overall Survival for Select Variables in Colorectal Cancer Patients

Supplementary Data 1: List of IRB Protocols Included In the Study

Supplementary Figure 1: Phase 1 Clinical Trials Patient Diagram

Supplementary Figure 2: (A) Progression Free Survival by Cohort (B) Overall Survival by Cohort

| 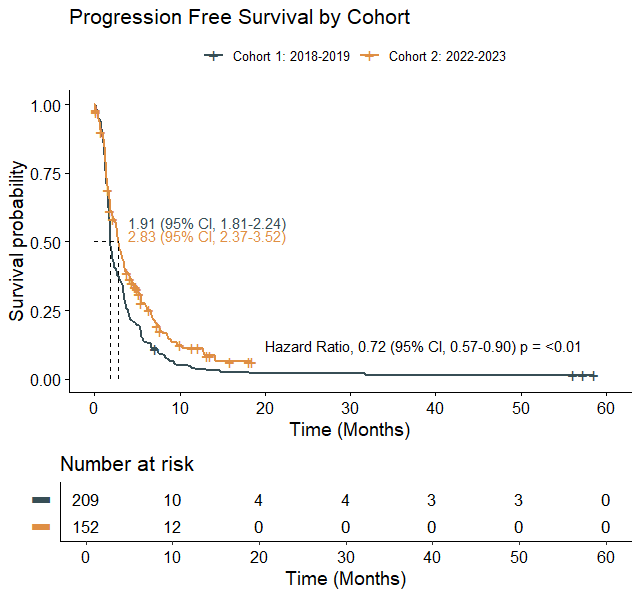 |
| --- |
| ***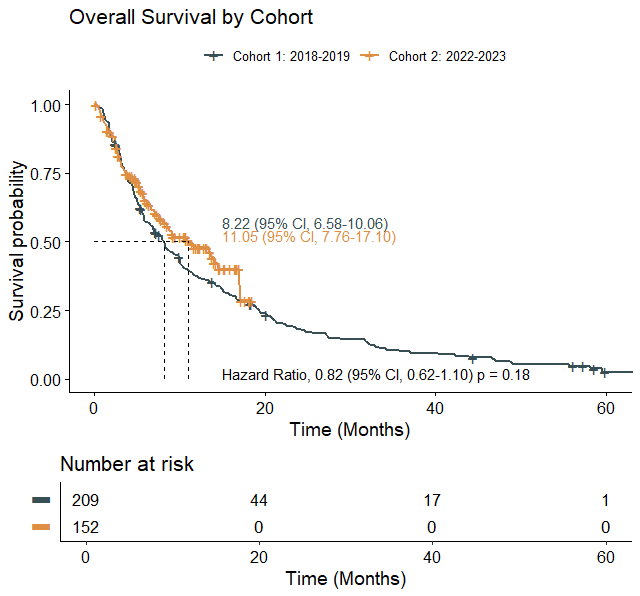*** |
| Dotted lines indicate median survival. Patients in Cohort 2 had a shorter follow up period due to being more recent. Patients who were neither off study nor lost to follow up were censored on 8/1/2023, our data collection cutoff date. Per graph (A), Patients in the post-intervention cohort had a prolonged median progression-free survival (PFS) of 2.83 months (95% Confidence Interval (CI) 2.37-3.52) compared to 1.91 months (95% CI 1.81-2.24). Median overall survival was 11.05 months (95% CI 7.76-17.10) post-intervention vs. 8.22 months (95% CI 6.58-10.06). |

Supplementary Figure 3: (A) Progression Free Survival by Cohort in CRC patients and (B) Overall Survival by Cohort in CRC Patients

| 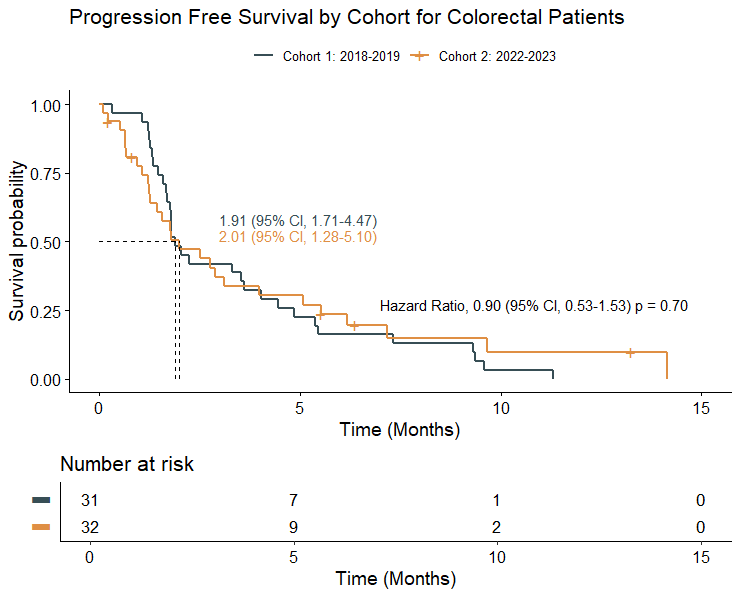 |
| --- |
| 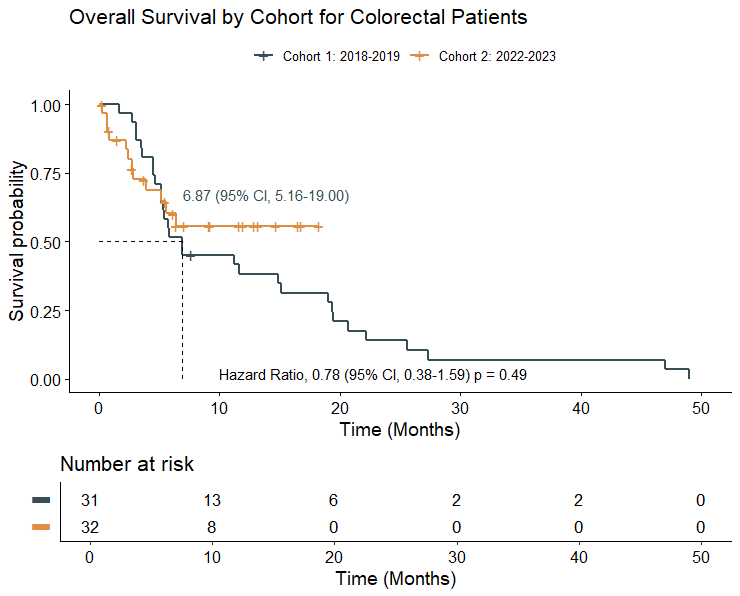 |
| Dotted lines indicate median survival. Patients in Cohort 2 had a shorter follow up period due to being more recent. Patients who were neither off study nor lost to follow up were censored on 8/1/2023, our data collection cutoff date. Median progression-free survival in the post-intervention cohort was 2.01 months (95% Confidence Interval (CI) of 1.28-5.10) vs. 1.91 months (95% CI 1.71-4.47) in the pre-intervention cohort. Median overall survival was 6.87 months (95% CI 5.16-19.00) pre-intervention. Median overall survival in the post-intervention cohort has not been reached.  Abbreviation: CRC: Colorectal cancer |

Supplementary Table 1: Median Progression-free Survival for Select Variables

| **Variable** | **No. of Patients (n = 361)** | **Median Progression Free Survival,**  **Months (95% CI)** |
| --- | --- | --- |
| Cohort |  |  |
| Cohort 1: 2018-2019 | 209 | 1.9 (1.8, 2.2) |
| Cohort 2: 2022-2023 | 152 | 2.8 (2.4, 3.5) |
| Race |  |  |
| White | 307 | 2.3 (1.9, 2.8) |
| Asian | 12 | 1.8 (1.4, —) |
| Black or African American | 5 | 2.2 (2.1, —) |
| More Than One Race | 4 | 2.3 (0.66, —) |
| Native Hawaiian or Other Pacific Islander | 1 | 2.7 (—, —) |
| Unknown / Not Reported | 32 | 1.8 (1.4, 2.9) |
| Ethnicity |  |  |
| NOT Hispanic or Latino | 327 | 2.1 (1.9, 2.7) |
| Hispanic or Latino | 33 | 2.2 (1.7, 3.1) |
| Insurance |  |  |
| Medicare | 165 | 2.6 (2.0, 3.1) |
| Medicaid | 161 | 2.7 (1.5, 3.3) |
| Private Insurance | 33 | 1.9 (1.8, 2.7) |
| Uninsured | 2 | 1.8 (1.8, —) |
| ADI (Halves) |  |  |
| 1-5 | 206 | 2.3 (2.0, 3.1) |
| 6-10 | 121 | 1.9 (1.7, 2.9) |
| Sex |  |  |
| Female | 189 | 2.0 (1.8, 2.7) |
| Male | 172 | 2.3 (1.9, 3.1) |
| ECOG |  |  |
| 0 | 158 | 2.6 (1.9, 3.4) |
| 1 | 198 | 2.0 (1.8, 2.7) |
| 2 | 5 | 1.6 (1.1, —) |
| Smoking Status |  |  |
| Non-smoker | 203 | 2.3 (1.9, 2.9) |
| Current Smoker | 13 | 2.7 (1.2, —) |
| Past Smoker | 145 | 1.9 (1.8, 2.7) |
| BMI |  |  |
| <18.5 | 28 | 1.8 (1.6, 2.7) |
| 18.5 - 24.9 | 149 | 2.0 (1.8, 2.9) |
| 25 - 29.9 | 115 | 2.2 (1.8, 3.4) |
| >=30 | 69 | 2.8 (1.9, 4.5) |
| Clinical Trial Treatment Type |  |  |
| Immunotherapy | 187 | 2.1 (1.8, 2.7) |
| Targeted Therapy | 104 | 1.9 (1.8, 2.9) |
| Antibody-drug Conjugate | 23 | 2.8 (1.6, 7.3) |
| Cytotoxic Therapy | 8 | 3.8 (2.9, —) |
| Combinations of Different Treatment Types | 39 | 3.3 (1.9, 4.5) |

Abbreviations: ADI: Area deprivation index. BMI: Body mass index. CI: Confidence interval. ECOG: Eastern Cooperative Oncology Group. Dashes in confidence intervals indicate that the value was not estimable due to limited number of events or heavy censoring.

Supplementary Table 2: Median Overall Survival for Select Variables

| **Variable** | **No. of Patients (n = 361)** | **Median Overall Survival, Months (95% CI)** |
| --- | --- | --- |
| Cohort |  |  |
| Cohort 1: 2018-2019 | 209 | 8.2 (6.6, 10) |
| Cohort 2: 2022-2023 | 152 | 11 (7.8, 17) |
| Race |  |  |
| White | 307 | 8.9 (7.9, 12) |
| Asian | 12 | 6.6 (3.4, —) |
| Black or African American | 5 | 11 (5.8, —) |
| More Than One Race | 4 | 27 (0.66, —) |
| Native Hawaiian or Other Pacific Islander | 1 | 4.8 (—, —) |
| Unknown / Not Reported | 32 | 5.3 (4.4, 14) |
| Ethnicity |  |  |
| NOT Hispanic or Latino | 327 | 8.6 (7.5, 11) |
| Hispanic or Latino | 33 | 8.3 (4.8, —) |
| Insurance |  |  |
| Medicare | 165 | 9.5 (7.9, 15) |
| Medicaid | 161 | 9.2 (5.0, 18) |
| Private Insurance | 33 | 7.9 (5.8, 10) |
| Uninsured | 2 | 4.8 (4.8, —) |
| ADI (Halves) |  |  |
| 1-5 | 206 | 10 (7.9, 14) |
| 6-10 | 121 | 8.2 (5.6, 12) |
| Sex |  |  |
| Female | 189 | 9.8 (8.1, 12) |
| Male | 172 | 7.5 (5.8, 12) |
| ECOG |  |  |
| 0 | 158 | 11 (8.7, 15) |
| 1 | 198 | 7.8 (5.6, 10) |
| 2 | 5 | 7.2 (3.2, —) |
| Smoking Status |  |  |
| Non-smoker | 203 | 10 (7.9, 14) |
| Current Smoker | 13 | 17 (3.8, —) |
| Past Smoker | 145 | 7.5 (5.8, 10) |
| BMI |  |  |
| <18.5 | 28 | 4.6 (3.6, 6.4) |
| 18.5 - 24.9 | 149 | 8.3 (6.9, 10) |
| 25 - 29.9 | 115 | 11 (7.9, 17) |
| >=30 | 69 | 12 (8.4, 19) |
| Clinical Trial Treatment Type |  |  |
| Immunotherapy | 187 | 9.2 (8.0, 14) |
| Targeted Therapy | 104 | 7.0 (5.7, 10) |
| Antibody-drug Conjugate | 23 | 9.8 (4.0, —) |
| Cytotoxic Therapy | 8 | 20 (12, —) |
| Combinations of Different Treatment Types | 39 | 6.3 (4.4, 21) |

Abbreviations: ADI: Area deprivation index. BMI: Body mass index. CI: Confidence interval. ECOG: Eastern Cooperative Oncology Group. Dashes in confidence intervals indicate that the value was not estimable due to limited number of events or heavy censoring.

Supplementary Table 3: Median Progression-free Survival for Select Variables in Colorectal Cancer Patients

| **Variable** | **No. of Patients (n = 66)** | **Median Progression Free Survival, Months (95% CI)** |
| --- | --- | --- |
| Cohort |  |  |
| 2018-2019 Trials | 32 | 2.0 (1.8, 4.0) |
| 2022-2023 Trials | 34 | 2.0 (1.3, 4.0) |
| Race |  |  |
| White | 57 | 2.0 (1.8, 4.0) |
| Asian | 3 | 1.9 (1.7, —) |
| Black or African American | 2 | 2.0 (1.8, —) |
| More Than One Race | 1 | 0.66 (—, —) |
| Unknown / Not Reported | 3 | 1.5 (1.3, —) |
| Ethnicity |  |  |
| NOT Hispanic or Latino | 60 | 2.0 (1.8, 3.6) |
| Hispanic or Latino | 6 | 1.6 (1.4, —) |
| Insurance |  |  |
| Medicare | 20 | 2.9 (1.7, 9.7) |
| Medicaid | 3 | 1.1 (0.66, —) |
| Private Insurance | 43 | 1.9 (1.8, 3.6) |
| ADI (Halves) |  |  |
| 1-5 | 44 | 2.8 (1.9, 5.1) |
| 6-10 | 19 | 1.7 (1.3, 3.6) |
| Clinical Trial Treatment Type |  |  |
| Immunotherapy | 45 | 1.9 (1.5, 4.0) |
| Targeted Therapy | 13 | 1.8 (1.6, —) |
| Combination Treatment | 8 | 5.0 (2.8, —) |

Abbreviations: ADI: Area deprivation index. CI: Confidence interval. Dashes in confidence intervals indicate that the value was not estimable due to limited number of events or heavy censoring.

Supplementary Table 4: Median Overall Survival for Select Variables in Colorectal Cancer Patients

| **Variable** | **No. of Patients (n = 66)** | **Median Survival, Months (95% CI)** |
| --- | --- | --- |
| Cohort |  |  |
| 2018-2019 Trials | 32 | 6.3 (5.2, 19) |
| 2022-2023 Trials | 34 | 9.2 (5.2, —) |
| Race |  |  |
| White | 57 | 11 (5.4, 19) |
| Asian | 3 | 4.7 (3.4, —) |
| Black or African American | 2 | 4.4 (3.1, —) |
| More Than One Race | 1 | 0.66 (—, —) |
| Unknown / Not Reported | 3 | 5.8 (3.6, —) |
| Ethnicity |  |  |
| NOT Hispanic or Latino | 60 | 6.9 (5.3, 19) |
| Hispanic or Latino | 6 | 6.4 (3.6, —) |
| Insurance |  |  |
| Medicare | 20 | 15 (5.3, —) |
| Medicaid | 3 | 3.1 (0.66, —) |
| Private Insurance | 43 | 6.4 (5.2, 19) |
| ADI (Halves) |  |  |
| 1-5 | 44 | 15 (6.4, 26) |
| 6-10 | 19 | 4.2 (2.8, 15) |
| Clinical Trial Treatment Type |  |  |
| Immunotherapy | 45 | 9.2 (5.6, 22) |
| Targeted Therapy | 13 | 4.5 (3.4, —) |
| Combination Treatment | 8 | 15 (2.9, —) |

Abbreviations: ADI: Area deprivation index. CI: Confidence interval. Dashes in confidence intervals indicate that the value was not estimable due to limited number of events or heavy censoring.

Supplementary Data 1: List of IRB Protocols Included In the Study

The following IRB-approved clinical trials were included in this study: 06-0155, 14-0151, 14-0251, 14-1055, 15-1135, 15-1354, 15-1579, 15-1581, 16-0207, 16-0628, 16-1057, 16-1089, 16-1333, 16-2248, 17-0074, 17-0256, 17-0339, 17-0402, 17-0948, 17-0991, 17-1154, 17-1452, 17-1547, 17-1652, 17-1745, 17-1909, 17-1943, 17-2198, 17-2381, 17-2450, 17-2451, 17-7807, 17-7809, 18-0033, 18-0154, 18-0187, 18-0685, 18-0965, 18-1150, 18-1263, 18-1526, 18-1659, 18-1672, 18-1721, 18-1816, 18-2417, 18-2529, 19-0539, 19-0604, 19-0946, 19-1458, 19-1727, 19-2761, 20-0216, 20-1289, 20-1690, 20-1845, 20-1994, 20-2042, 20-2818, 20-2976, 20-3038, 21-3286, 21-3431, 21-3737, 21-4026, 21-4586, 21-4633, 21-4716, 21-4887, 22-0094, 22-0228, 22-0318, 22-0359, 22-0510, 22-0877, 22-1125, 22-1188, 22-1245, 22-1490
